# DCX^+^ neuronal progenitors contribute to new oligodendrocytes during remyelination in the hippocampus

**DOI:** 10.1038/s41598-020-77115-w

**Published:** 2020-11-18

**Authors:** Barbara Klein, Heike Mrowetz, Christina Kreutzer, Peter Rotheneichner, Pia Zaunmair, Simona Lange, Roland Coras, Sebastien Couillard-Despres, Francisco J. Rivera, Ludwig Aigner

**Affiliations:** 1 Institute of Molecular Regenerative Medicine, Paracelsus Medical University, Salzburg, Austria; 2 Spinal Cord Injury and Tissue Regeneration Center Salzburg (SCI-TReCS), Paracelsus Medical University, Salzburg, Austria; 3 Institute of Experimental Neuroregeneration, Paracelsus Medical University, Salzburg, Austria; 4 Department of Neuropathology, Universitätsklinikum Erlangen, Friedrich-Alexander University Erlangen-Nürnberg (FAU), Erlangen, Germany; 5 Austrian Cluster for Tissue Regeneration, Vienna, Austria; 6 Laboratory of Stem Cells and Neuroregeneration, Faculty of Medicine, Institute of Anatomy, Histology and Pathology, Universidad Austral de Chile, Valdivia, Chile; 7 Center for Interdisciplinary Studies on the Nervous System (CISNe), Universidad Austral de Chile, Valdivia, Chile

**Keywords:** Gliogenesis, Myelin biology and repair, Neurogenesis, Stem cells in the nervous system

## Abstract

A pool of different types of neural progenitor cells resides in the adult hippocampus. Apart from doublecortin-expressing (DCX^+^) neuronal progenitor cells (NPCs), the hippocampal parenchyma also contains oligodendrocyte precursor cells (OPCs), which can differentiate into myelinating oligodendrocytes. It is not clear yet to what extent the functions of these different progenitor cell types overlap and how plastic these cells are in response to pathological processes. The aim of this study was to investigate whether hippocampal DCX^+^ NPCs can generate new oligodendrocytes under conditions in which myelin repair is required. For this, the cell fate of DCX-expressing NPCs was analyzed during cuprizone-induced demyelination and subsequent remyelination in two regions of the hippocampal dentate gyrus of DCX-CreER^T2^/Flox-EGFP transgenic mice. In this DCX reporter model, the number of GFP^+^ NPCs co-expressing Olig2 and CC1, a combination of markers typically found in mature oligodendrocytes, was significantly increased in the hippocampal DG during remyelination. In contrast, the numbers of GFP^+^PDGFRα^+^ cells, as well as their proliferation, were unaffected by de- or remyelination. During remyelination, a higher portion of newly generated BrdU-labeled cells were GFP^+^ NPCs and there was an increase in new oligodendrocytes derived from these proliferating cells (GFP^+^Olig2^+^BrdU^+^). These results suggest that DCX-expressing NPCs were able to contribute to the generation of mature oligodendrocytes during remyelination in the adult hippocampus.

## Introduction

The adult hippocampus contains at least two different types of progenitor cells, i.e. neuronal progenitor cells (NPCs) and oligodendrocyte progenitor cells (OPCs). It is not entirely clear to what extent the identity as well as the fate and functions of these progenitors overlap, in particular during pathological processes (for a review see^1^). NPCs reside in the subgranular zone (SGZ) of the dentate gyrus (DG), one of two prominent regions of neurogenesis in the adult brain. During hippocampal neurogenesis, the radial glia-like neural stem cells in the SGZ generate a pool of amplifying progenitors that are generally committed to a neuronal fate. These cells give rise to doublecortin-expressing (DCX^+^) NPCs which migrate and differentiate into newly generated granule neurons that functionally integrate into the granular layer (GL) of the hippocampal DG (for a review see^2,3^). Due to its expression pattern, DCX has become a widely used marker for the analysis of adult neurogenesis^4,5^. Imaging and fate mapping tools based on the DCX promoter have been developed and successfully implemented in neurogenesis research^6–8^.

While proliferating NPCs seem to reside exclusively in neurogenic regions, OPCs are distributed throughout the CNS parenchyma and are able to give rise to mature oligodendrocytes, which are responsible for myelination of axons (for a review see^9^). OPCs are characterized by the expression of oligodendrocyte transcription factor 2 (Olig2), platelet-derived growth factor receptor α (PDGFRα), and the proteoglycan NG2 (for a review see^10^). Mature oligodendrocytes can be identified by the co-expression of Olig2 and CC1^11^.

The hippocampus contains various populations of myelinated axons. For example, parvalbumin-positive interneurons have myelinated axons^12^. Also, the hippocampus is connected to cortical and subcortical regions via the perforant pathway and the extent of myelination in this region plays a role in cognitive functions and diseases such as Alzheimer’s disease and epilepsy (for a review see^13^). However, there are only few studies focusing on myelin repair in this region. This is surprising since in multiple sclerosis (MS) patients, the hippocampus is often severely affected by demyelination, microstructural damage, altered connectivity, and atrophy, which may lead to an impairment of episodic memory^14–18^. In particular, the CA4/DG subfield is the first region of the hippocampus that is atrophied during early MS stages^19^, emphasizing that this hippocampal subregion deserves special attention in MS research.

The copper-chelating substance cuprizone reproducibly induces cell death in oligodendrocytes and thus leads to demyelination, which is followed by spontaneous myelin repair^20–22^, in various brain regions including also the hippocampus^23–27^. Therefore, cuprizone treatment is widely used as a model to mimic a central event of MS pathology, i.e. demyelination. This is in contrast to the experimental autoimmune encephalomyelitis (EAE) model for MS, in which the hippocampus is not demyelinated^28^. Besides structural changes, chronic treatment with cuprizone alters functional connectivity in the hippocampus, in particular in the DG^29^. Nickel and colleagues described that a 5-week cuprizone treatment led to myelin loss in both in the GL and the hilus of the hippocampal DG, which recovered 2 weeks after cuprizone withdrawal^28^. However, the process of remyelination in the hippocampus, more specifically the cellular origin of new oligodendrocytes in the DG is rather unexplored and might differ from what has been described in white matter regions in which no NPCs reside.

Apart from their role in adult neurogenesis, DCX^+^ NPCs have been found to migrate from the subventricular stem cell niche to demyelinated lesions in the corpus callosum^30^, and fate mapping analysis showed that they subsequently expressed markers of mature oligodendrocytes^31^. It is still unclear, however, if this oligodendroglial fate is also possible for DCX^+^ NPCs derived from the hippocampal stem cell niche. For this reason, the aim of the present study was to investigate whether DCX^+^ NPCs are able to replace lost oligodendrocytes in the hippocampal DG after cuprizone-induced demyelination.

## Material and methods

### Animals and cuprizone treatment

Wildtype 2-months-old male C57BL/6J mice were obtained from Janvier. These animals were divided into four treatment groups (n = 6 per group, 24 mice in total). To induce demyelination, mice received standard food pellets pre-mixed with 0.2% cuprizone (ssniff Spezialdiäten) for 6 weeks. Afterwards, during the remyelination period, a normal diet consisting of standard food pellets was resumed for 1 or 2 weeks. Control animals received standard food pellets (ssniff Spezialdiäten) for 8 weeks.

For cell fate analysis of DCX-expressing cells, transgenic DCX-CreER^T2^/Flox-EGFP mice were used^8^. At the age of 3 months, these mice were injected with tamoxifen (i.p., 100 mg/kg bodyweight daily) during the first 5 days of the experiment (Day 1 to 5) to induce permanent expression of EGFP in DCX-expressing cells of DCX-CreER^T2^/flox-EGFP mice. After tamoxifen treatment, mice were divided into four groups. The first group (control 1 week; n = 10) was perfused 10 days after the last administration of tamoxifen (on Day 15) to analyze the starting population of GFP-labeled cells. The second group (control group; n = 8) received normal chow for 6 weeks. The third group (demyelination group; n = 8) received food pellets pre-mixed with 0.2% cuprizone for 6 weeks. Finally, the fourth group (remyelination group; n = 7) also received cuprizone for 6 weeks to induce demyelination, and afterwards normal chow was given for 2 weeks to allow for remyelination. For labeling of proliferating cells, BrdU was injected i.p. (50 mg/kg body weight, in PBS) on 5 consecutive days. Group 1 received BrdU at the beginning of the second week (Day 8–12), groups 2 and 3 were treated with BrdU at the beginning of week 6 and perfused 3 days after the last injection. Group 4 was injected with BrdU at the onset of cuprizone withdrawal and survived for 10 more days.

Mice were housed in the animal facility of the Paracelsus Medical University in Salzburg, Austria under standard conditions including a 12-h light/dark cycle and water and food ad libitum. All experiments were executed in accordance with relevant national and international guidelines and regulations, in particular, the Directive (2010/63/EU) of the European Parliament and of the Council/European Communities Council Directive of 24 November 1986 (86/609/EEC). All experimental protocols were approved by the Federal State Government Salzburg, Austria under permit number 20901-TVG/61/14-2016.

### Tissue preparation

Tissue fixation was performed as previously detailed elsewhere^5^. Terminal anesthesia was administered via i.p. injection: Ketamine (273 mg/kg body weight), xylazine (71 mg/kg body weight) and acepromazine (4 mg/kg body weight) in a physiological saline solution. Then anesthetized mice were transcardially perfused with a 0.9% NaCl (w/v) solution followed by 4% paraformaldehyde in PBS (pH 7.4). After extraction, brain tissue was postfixed overnight, immersed in 30% sucrose solution (w/v, in PBS) for 48 h, and then processed on dry ice into 40 µm-thick sagittal sections using a Leica SM 2000R sliding microtome. The resulting sections were separated into representative tenths of each hemisphere. That means every tenth section was put into a different tube containing a cryoprotection solution (equal parts glycerin, ethylene glycol, 0.2 M phosphate buffer pH 7.4, and H_2_O) and stored at − 20 °C.

### Immunohistochemistry

The protocol used for immunohistological stainings was previously described in detail^32^. An antigen retrieval step was added to the protocol after the first washing of the tissue sections. For antigen retrieval, the sections were incubated in 10 mM citrate buffer (pH 6.0) in a steamer at 100 °C for 20 min, followed by 3 × 5 min washing in PBS. For PDGFRα, the antigen retrieval was shortened, i.e. the hot citrate buffer was put on the sections only for 10 min at RT. A tenth of a hemisphere was used for each immunohistological staining.

The following antibodies and dilutions were used: Primary antibodies: rat anti-MBP (1:150, MCA409S, AbD Serotec), rabbit anti-Olig2 (1:300, AB9610, Millipore), mouse anti-APC (CC1) (1:500, D00123356, Calbiochem), mouse anti-PCNA (1:500, A1111, Santa Cruz), rabbit anti-DCX (1:200, 4606, Cell Signaling), chicken anti-GFP (1:500, A10262, Life Technologies), rabbit anti-NG2 (1:200, AB5220, Millipore), rabbit anti-PDGFRα (1:500, 3174, Cell Signaling), rat anti-BrdU (1:500, OBT0030, Serotec), and mouse anti-APC (CC1) (1:500, OP80, Calbiochem). Secondary antibodies (all diluted 1:1000): rabbit anti-rat biotinylated (BA-4001, VectorLab), goat anti-rat Alexa 568 (11077, Mol Probes), donkey anti-mouse Alexa 488 (A21202, Invitrogen), donkey anti-rabbit Alexa 568 (A10042, Life Tech), donkey anti-rabbit Alexa 405 (ab175651, Abcam; Figs. 7, 8), donkey anti-mouse Alexa 647 (715-605-151, Jackson), donkey anti-chicken Alexa 488 (703–545-135, Dianova), donkey anti-chicken Alexa 488 (703845155, Jackson; Figs. 7, 8), and donkey anti-mouse Alexa 647 (715605151, Jackson; Figs. 7, 8). Cell nuclei were stained with 4′,6-diamidino-2-phenylindole dihydrochloride at a concentration of 0.5 μg/μl (DAPI; Sigma-Aldrich).

### Microscopy and image processing

The MBP staining was imaged with a Zeiss Axioplan microscope (5× objective) using the 4.30.01 version of the AxioCam MRC5 Zeiss Imaging Software. Fluorescent images were acquired with a Zeiss LSM 700 confocal scanning laser microscope with the Zen 2012 LSM software. Z-stacks across the whole 40 µm section thickness were taken using a 20× objective and 0.5× zoom. For the figures, orthogonal maximum intensity projections were created with the Zen software, and, if required, brightness and contrast were adjusted for the whole image.

### Quantitative analysis of immunohistochemistry

All image analysis was done blinded (i.e. without knowing group or mouse number) using the software ImageJ 1.48 (GNU General Public License). The percentage area covered by the MBP staining was determined using the “Analyze particles” function of ImageJ. For each mouse, four tissue sections were used to analyze staining in the two target regions. The bright-field microscopic images of MBP staining were quantified in an area of 0.014 mm^2^ in the hilus of the dorsal hippocampal DG and an area of 0.1 mm^2^ in the GL plus SGZ.

For analysis of cell numbers in confocal images of the dorsal hippocampal DG, the four sections with the biggest hilus area within a tenth of a hemisphere were analyzed (approximate range of ML coordinates for the analyzed sections: 0.48–2.52 mm lateral). The SGZ was defined as the first layer beneath the GL plus one cell layer below that. The total cell numbers within the regions of interest were counted using the “Cell Counter” plug-in (cell_counter.jar, version 2, GNU General Public License) for ImageJ and then multiplied per 10. For analysis of the CC, the same method was used for the region of the CC that was located directly above the hippocampal DG.

### Statistical analysis

Data are shown as means + standard deviation (S.D.). All statistical analysis was done using Prism 7 (Graphpad Software Inc). Data were tested for normality using a Shapiro–Wilk normality test. Statistical significance for normally distributed data was determined with a one-way ANOVA followed by a Tukey post-hoc test in which each group was compared to all others. For not-normally distributed data, statistical significance was determined using a Kruskal–Wallis test followed by a Dunn’s post-hoc test in which each group was compared to all others. The resulting p-values are represented as: *p < 0.05, **p < 0.01, and ***p < 0.001.

## Results

### The hippocampal DG responds to cuprizone treatment with de- and remyelination

**Figure 1:**
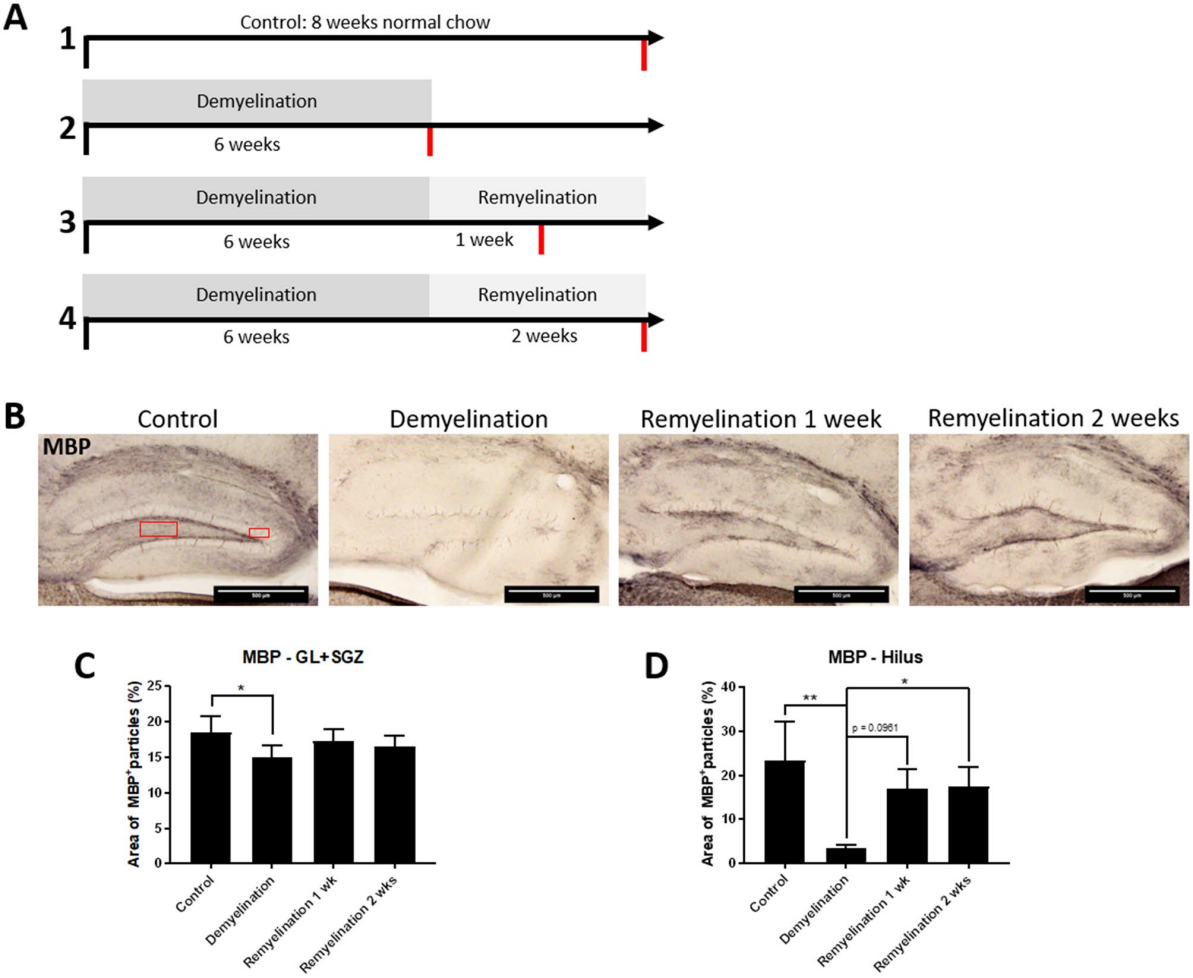
Experimental design and analysis of de- and remyelination in the dorsal hippocampal DG in wildtype mice. (**A**) Overview of the experimental setup and the four treatment groups (controls, demyelination, 1-week remyelination, and 2-weeks remyelination). Time of tissue collection is indicated by red lines. (**B**) Myelination in the hippocampal DG was analyzed by MBP staining. The rectangles indicate the regions of interest: Hilus and granular layer (GL) plus subgranular zone (SGZ). (**C**) In the GL plus SGZ, a significant demyelination after cuprizone treatment was observed. (**D**) In the hilus of the dorsal hippocampal DG, the myelin changes were more pronounced. Values are shown as means + S.D. (n = 6 per group). Statistical significance was evaluated using (**C**) a one-way ANOVA followed by a Tukey post-hoc test or (**D**) a Kruskal–Wallis test followed by a Dunn’s post-hoc test. The p-values are indicated in the graphs: *p < 0.05, and **p < 0.01. Bars: (**B**) 500 µm.

First, we assessed whether cuprizone induced de- and remyelination in the dorsal hippocampal DG of wild-type mice. The experimental groups were: (1) controls (mice receiving normal chow), (2) demyelination (after a 6-week period of cuprizone treatment via chow), (3) 1 week remyelination (as group 2, but with one additional week without cuprizone), and (4) 2 week remyelination (as group 2, but with two additional weeks without cuprizone) (experimental setup see Fig. 1A). Myelin was detected by MBP immunostaining (Fig. 1B). As expected, since they are composed of densely packed neuronal cell bodies, cuprizone-induced myelin changes in the GL and SGZ were subtle. Nevertheless, MBP levels were significantly lower in the demyelination group than in controls in this region (Fig. 1B,C). In the hilus of the hippocampal DG, which contains more myelinated axons, a pronounced demyelination was observed after cuprizone treatment, which was followed by a remarkable remyelination after 2 weeks (Fig. 1B,D).

**Figure 2:**
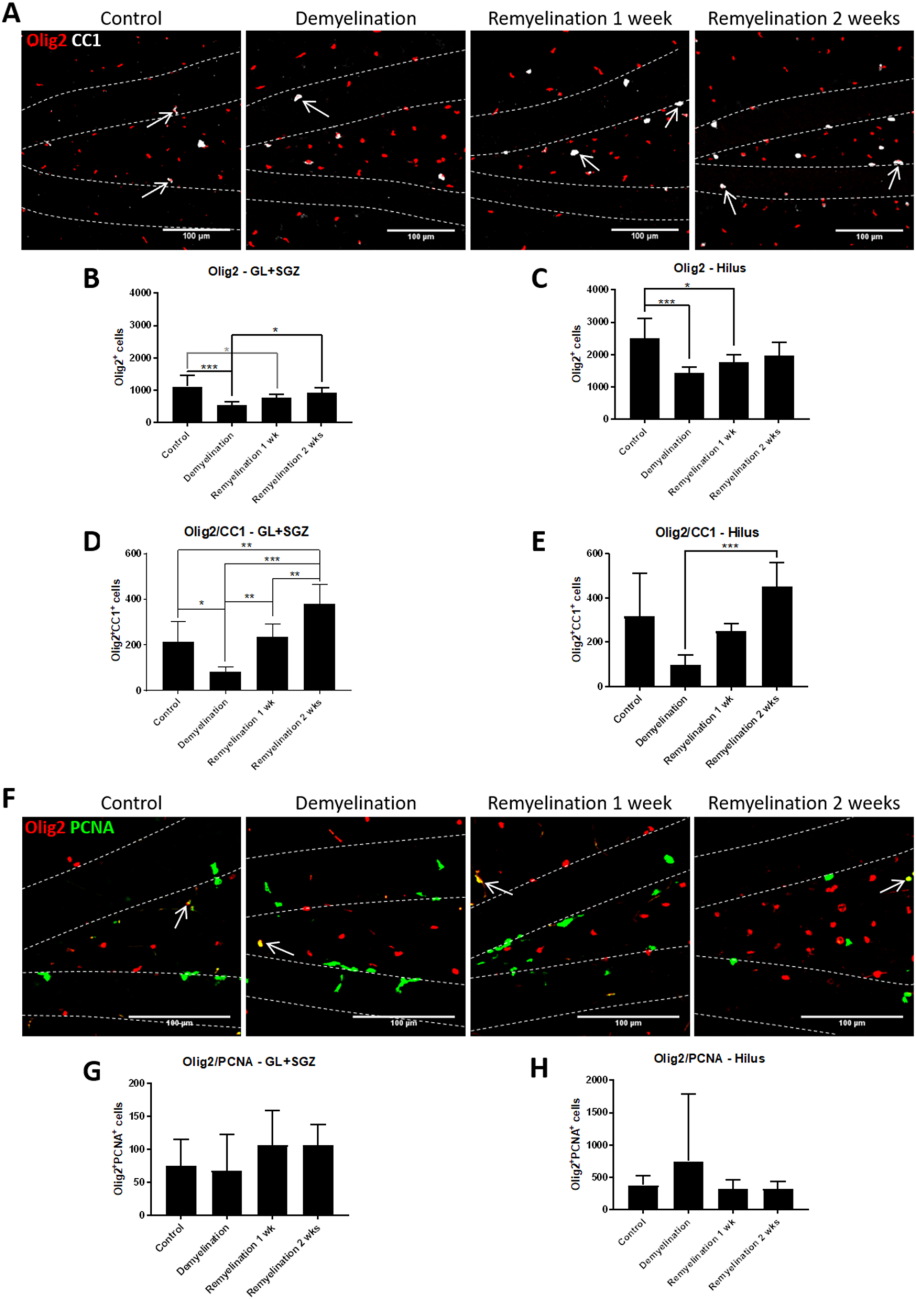
Restored numbers of oligodendrocyte lineage cells during remyelination in wildtype mice. (**A**) Double-labeling of Olig2 (red), a marker for oligodendrocyte lineage cells, and CC1 (white) which is expressed by mature oligodendrocytes. Double-positive cells are indicated by arrows. The white dashed line traces the outline of the GL. (**B**) In the GL plus SGZ, and (**C**) the hilus of the dorsal hippocampal DG, the numbers of Olig2^+^ cells were significantly decreased during demyelination and the first week of remyelination. Afterwards, the numbers were restored to control levels. (**D**) In the GL plus SGZ and (**E**) the hilus, Olig2^+^CC1^+^ cells were also significantly reduced after demyelination, but they already returned to control levels after the first week of remyelination, and exceeded those 1 week later (only significant in GL plus SGZ). (**F**) Double-labeling of Olig2 (red) and PCNA (green) which is expressed in proliferating cells. Double-positive cells are indicated by arrows. In the (**G**) GL plus SGZ and (H) in the hilus of DG, de- or remyelination had a no significant effect on the numbers of proliferating oligodendrocyte lineage cells. Values are depicted as means + S.D. (n = 6 per group). Statistical significance was evaluated using (**B**–**D**,**G**) a one-way ANOVA followed by a Tukey post-hoc test or (**E**,**H**) a Kruskal–Wallis test followed by a Dunn’s post-hoc test. The p-values are indicated in the graphs: The p-values are indicated in the graphs: *p < 0.05, **p < 0.01, and ***p < 0.001. Bars: (**A**,**F**) 100 µm.

The temporal pattern of de- and remyelination in the hippocampus correlated with the loss and re-appearance of cells of the oligodendroglial lineage (Olig2^+^) and of mature oligodendrocytes (Olig2^+^CC1^+^) (Fig. 2A). In both regions analyzed, demyelination led to a significant decrease of Olig2^+^ cells. After the second week of remyelination, the Olig2^+^ numbers returned to control levels (Fig. 2B,C). For Olig2^+^CC1^+^ mature oligodendrocytes, a similar pattern was observed. Even though the numbers of total Olig2^+^ oligodendroglial cells were approx. 2.5 times lower in the GL plus SGZ than in the hilus under control conditions, the numbers of mature Olig2^+^CC1^+^ oligodendrocytes were restored to similar levels in both regions during remyelination. Remarkably, the numbers of mature oligodendrocytes even significantly exceeded control levels in GL plus SGZ after the second week of remyelination (Fig. 2D,E). The number of proliferating cells of the oligodendrocyte lineage was analyzed by co-labeling of Olig2 with proliferating cell nuclear antigen (PCNA) (Fig. 2F). The number of Olig2^+^PCNA^+^ cells was approximately 5–10 times higher in the hilus than in the GL plus SGZ. Only in the hilus there was a trend towards higher numbers of Olig2^+^PCNA^+^ during demyelination, otherwise neither de- nor remyelination had a significant effect (Fig. 2G,H).

To summarize, we found a significant cuprizone-induced depletion of oligodendroglial cells and demyelination, which was followed by spontaneous recovery upon cuprizone withdrawal, in the hippocampus. The robust restoration of numbers of mature oligodendrocytes in the GL plus SGZ was unexpected, since this region not only contains fewer myelinated axons than the hilus, but also fewer OPCs, which, moreover, proliferate less in response to demyelination. Next, we took a closer look at the other progenitor type, NPCs, which derive from the stem cell niche in the SGZ and might be a source for the generation of oligodendrocytes.

### Fate analysis of DCX-expressing cells: GFP^+^ NPCs co-express oligodendroglial markers in DG subregions

**Figure 3:**
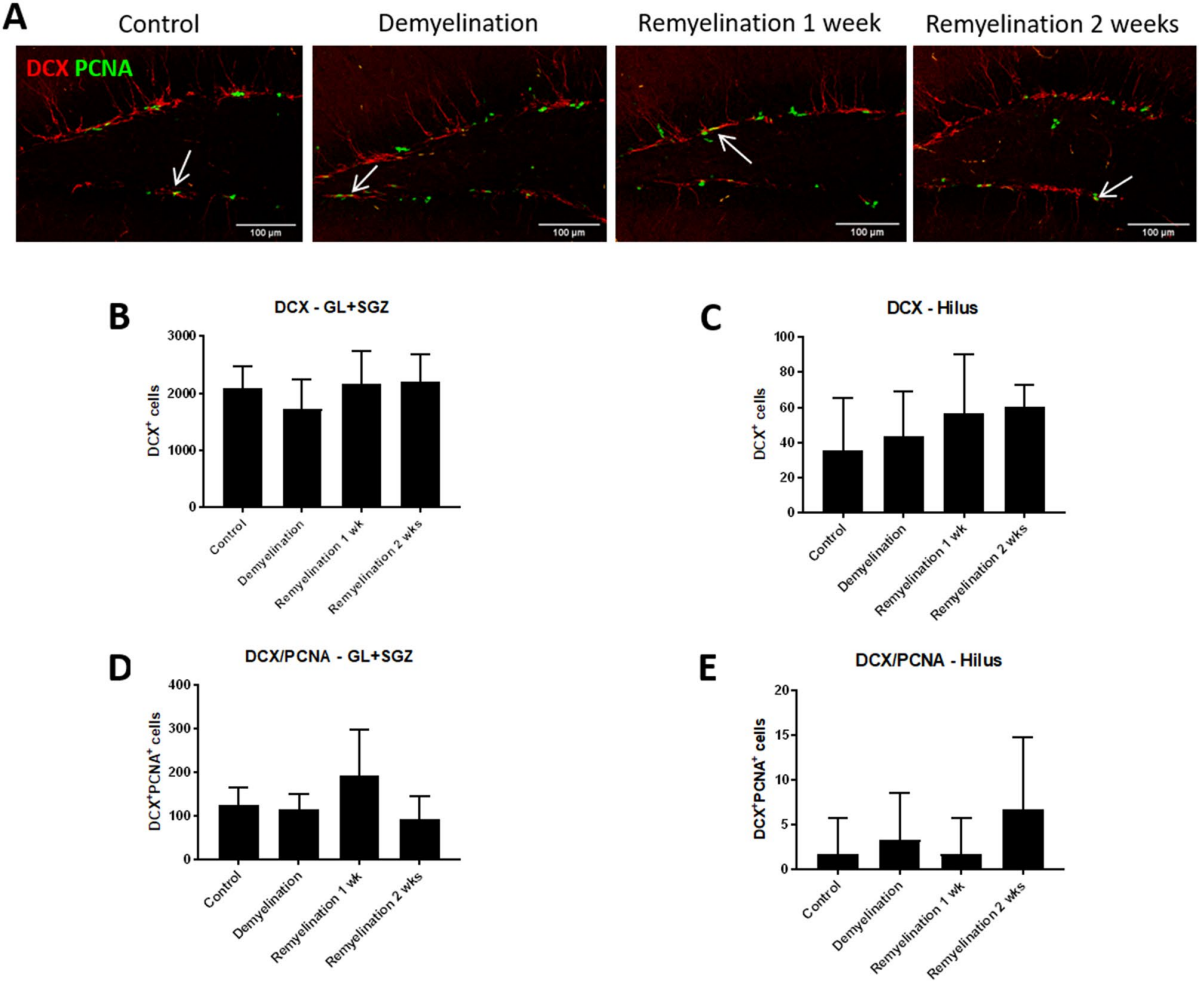
Proliferation of DCX^+^ NPCs unchanged after cuprizone treatment. (**A**) Double-labeling of DCX (red), a marker for NPCs, and PCNA (green) which is expressed in proliferating cells. Double-positive cells are indicated by arrows. (**B**) In the GL plus SGZ and (**C**) the hilus of the hippocampal DG, de- and remyelination did not significantly affect the number of DCX^+^ cells in either region. (**D**) The proliferation of DCX^+^ cells in the GL plus SGZ was not affected by treatment with cuprizone. (**E**) In the hilus, only few DCX^+^PCNA^+^ cells were observed, and de- or remyelination did not have significant effects. Values are depicted as means + S.D. (n = 6 per group). Statistical significance was evaluated using (**B**-**D**) a one-way ANOVA followed by a Tukey post-hoc test or (**E**) a Kruskal–Wallis test followed by a Dunn’s post-hoc test. No significant differences were found (at the p < 0.05 level). Bars: (**A**) 100 µm.

In agreement with the neurogenic zone being located in the SGZ, the numbers of DCX^+^ NPCs were much higher in the GL plus SGZ than in the hilus (Fig. 3A). De- and remyelination did not significantly affect the total number of these cells or their proliferation in either region (Fig. 3B–E).

**Figure 4:**
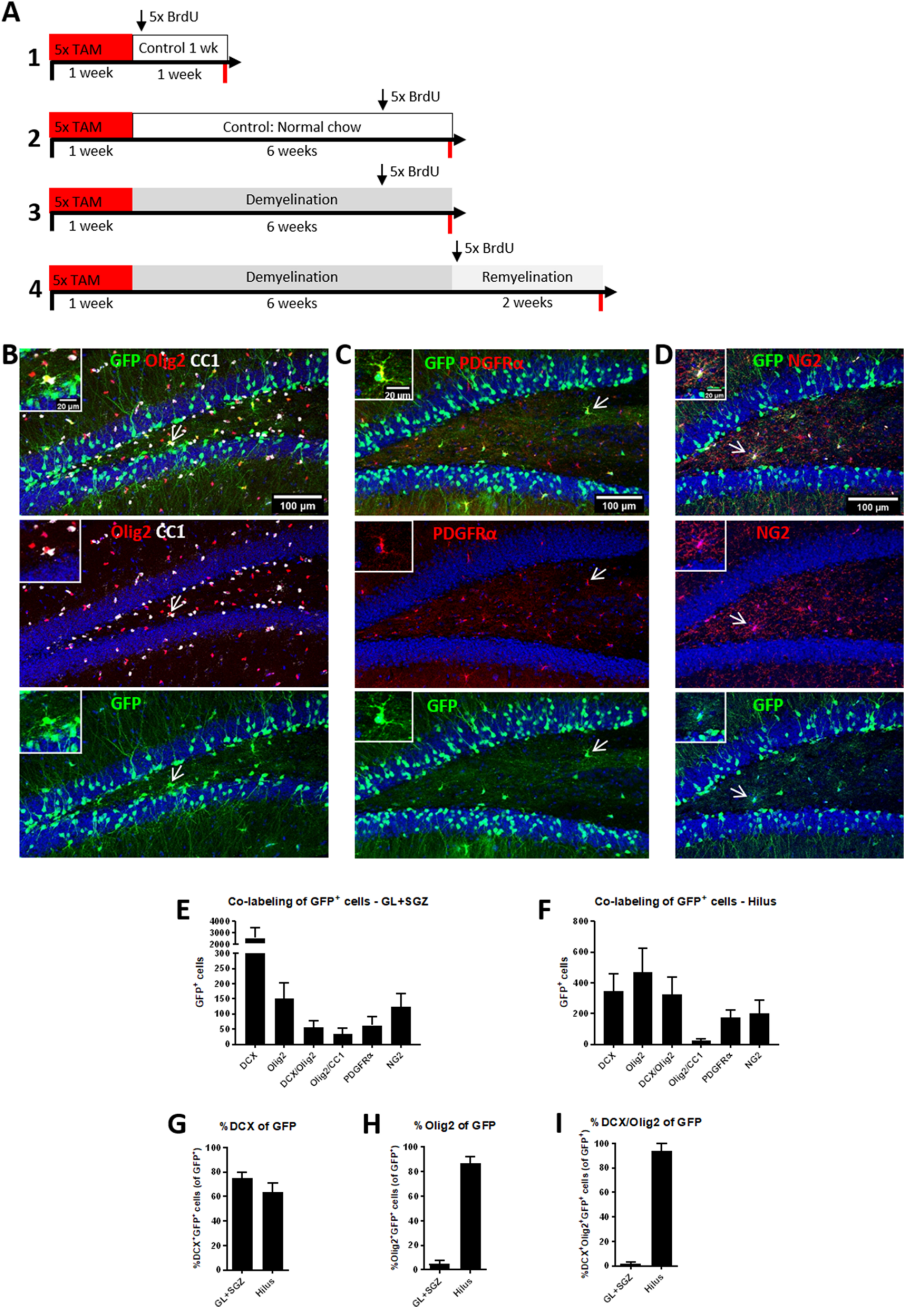
GFP-labeled cell populations 1 week after tamoxifen induction. (**A**) Overview of the timeline of the experiment with transgenic DCX-CreER^T2^/Flox-EGFP mice and the four treatment groups (control 1 week, control, demyelination, and 2 weeks remyelination). The period of tamoxifen treatment to induce expression of the GFP reporter gene is shown by the horizontal red bars (5 daily injections at the beginning of the first week). BrdU injections to label proliferating cells are represented by a vertical arrow. Time of tissue collection is indicated by red lines. (**B**) Triple-labeling of GFP (green), Olig2 (red) a marker for oligodendrocyte lineage cells, and CC1 (white) which is expressed by mature oligodendrocytes in the 1-week control group (10 days after the last TAM injection). The GFP^+^Olig2^+^CC1^+^ cell which is indicated by arrows is magnified in the inserts in the upper left corners. (**C**) Double-labeling of GFP (green) and PDGFRα (red) a marker for OPCs. The GFP^+^PDGFRα^+^ cell which is indicated by arrows is magnified in the inserts in the upper left corners. (**D**) Double-labeling of GFP (green) and NG2 (red). The GFP^+^NG2^+^ cell which is indicated by arrows is magnified in the inserts in the upper left corners. (**E**,**F**) Analysis of the different GFP^+^ cell populations directly after tamoxifen induction (control group 1 week; n = 10). The total numbers of GFP^+^ cells which were DCX^+^, Olig2^+^, DCX^+^Olig2^+^, Olig2^+^CC1^+^, PDGFRα^+^ or NG2^+^ are shown for (**E**) GL plus SGZ and (**F**) the hilus of the DG. The percentages of GFP^+^ cells in which also (**G**) the DCX protein or (**H**) the Olig2 protein or (**I**) both were detected by antibody staining in the GL plus SGZ or hilus. Values are depicted as means + S.D. Bars: (**A**–**C**) 100 µm, inserts 20 µm.

Next, we analyzed if DCX^+^ cells contribute to replacing lost oligodendrocytes by changing their default differentiation pattern. To find out whether DCX-expressing NPCs give rise to part of the oligodendroglial cell pool, transgenic DCX-CreER^T2^/Flox-EGFP mice were used (experimental setup see Fig. 4A). The first step was to characterize the cell population labeled by the GFP reporter at the beginning of the experiment. Additionally, it was assessed if these DCX reporter GFP^+^ cells co-expressed oligodendroglial markers (Olig2, CC1, PDGFRα, and NG2) directly after the end of the reporter gene induction period (control 1 week; Fig. 4B–I). The total numbers of GFP^+^ cells which co-expressed these different markers are shown for GL plus SGZ (Fig. 4E) and hilus (Fig. 4F).

In both DG subregions analyzed, the majority of GFP^+^ cells were also labeled by an antibody detecting the DCX protein (GL plus SGZ: 75 ± 5%, hilus: 64 ± 7% of all GFP^+^ cells, Fig. 4G), confirming that the reporter targeted the right cell population. In the GL plus SGZ, a small portion of GFP^+^ cells were labeled by an Olig2 antibody (5 ± 3% of all GFP^+^ cells), a marker of the oligodendroglial lineage (Fig. 4H). The percentage of GFP^+^Olig2^+^ cells was considerably higher (87 ± 5%) in the hilus of the DG (Fig. 4H). Also, the percentage of GFP^+^ DCX^+^ cells which were also positive for Olig2 differed greatly between the two regions (GL plus SGZ: 2 ± 1%, hilus: 94 ± 6% of GFP^+^ DCX^+^ cells, Fig. 4I). Surprisingly, a small number of DCX reporter GFP^+^ cells had the marker profile of mature oligodendrocytes (Olig2^+^CC1^+^) in both regions (Fig. 4E,F). The number of GFP^+^ cells which were positive for PDGFRα or NG2 was similar to the number of GFP^+^Olig2^+^ cells in both DG subregions analyzed (Fig. 4E,F).

These data suggest that, under control conditions, a very small portion of NPCs in the DG differentiate to mature oligodendrocytes. Moreover, these two subregions of the hippocampal DG might contain different subpopulations of DCX^+^ NPCs, which seem to be more likely to co-express oligodendroglial markers in the hilus.

### GFP^+^ NPCs increasingly express oligodendroglial markers during de- and remyelination

**Figure 5:**
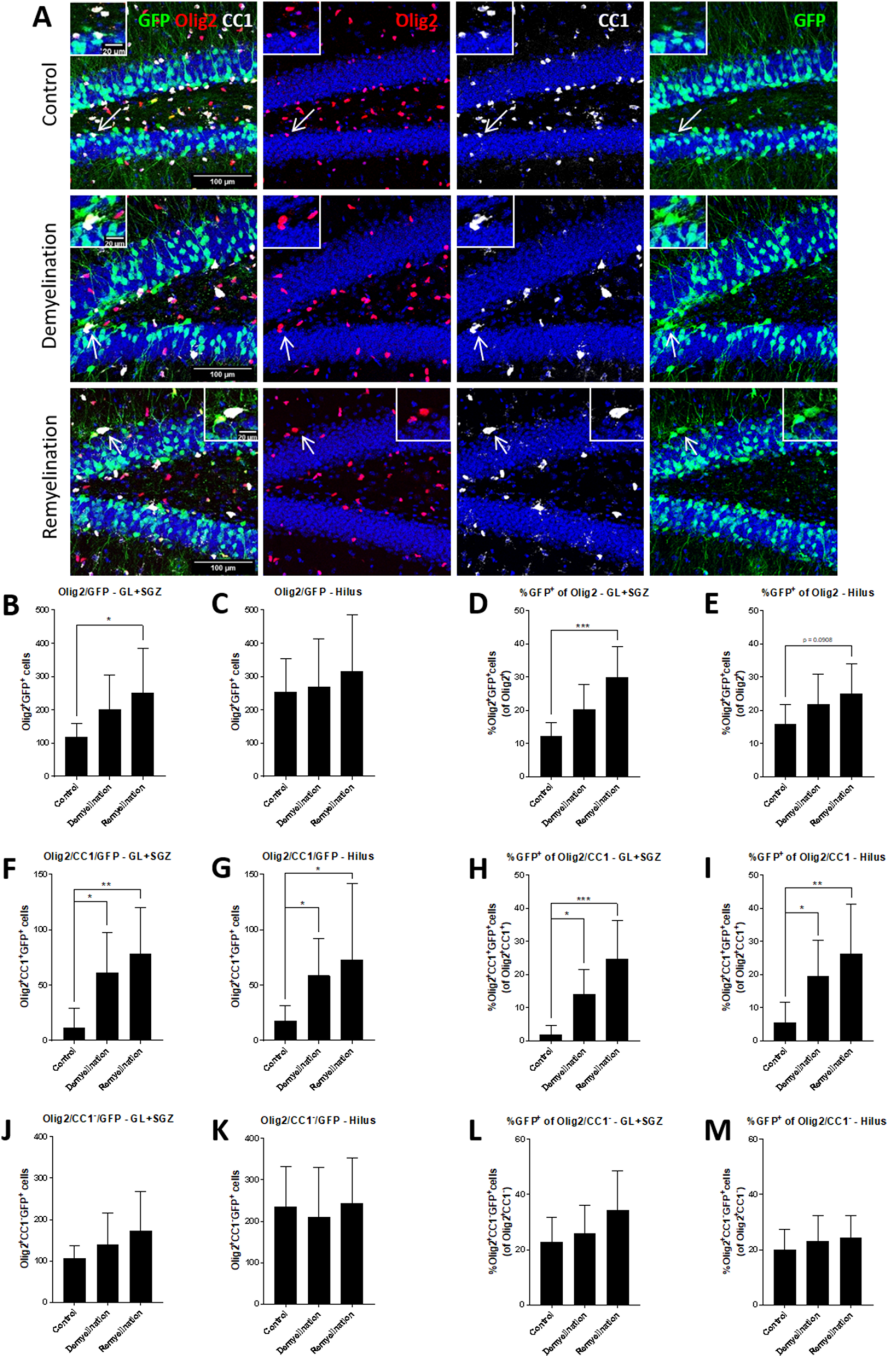
Increased numbers of GFP^+^Olig2^+^CC1^+^ mature oligodendrocytes during remyelination in the hippocampal DG. (**A**) Triple-labeling of GFP (green), Olig2 (red) a marker for oligdendrocyte lineage cells, and CC1 which is expressed by mature oligodendrocytes (white) in controls, the demyelination group, and after 2 weeks of remyelination. The GFP^+^Olig2^+^CC1^+^ cells which are indicated by arrows are magnified in the inserts in the corners. (**B**,**C**) In the DCX reporter model, the numbers GFP^+^Olig2^+^ cells were significantly increased in the GL plus SGZ after 2 weeks of remyelination (**B**), but not in the hilus (**C**). (**D**,**E**) Also the percentage of GFP-expressing Olig2^+^ cells was significantly higher in the remyelination group in the GL plus SGZ (**D**), and a similar trend (p = 0.0908) was observed for the hilus (**E**). (**F**,**G**) The increase of GFP^+^Olig2^+^CC1^+^ cells during de- and remyelination was significant in the GL plus SGZ (**F**) and in the hilus (**G**), and a similar pattern was observed for the (**H**,**I**) percentage of GFP-expressing mature oligodendrocytes. (**J**,**M**) GFP^+^Olig2^+^CC1^−^ OPCs and the percentage of these cells expressing GFP were not significantly increased in both regions. Values are depicted as means + S.D. (controls and demyelination: n = 8 per group; remyelination: n = 7). Statistical significance was evaluated using (**E**, **I**, **K**, **M**) a one-way ANOVA followed by a Tukey post-hoc test or (**B**–**D**, **F**-**H**, **J**, **L**) a Kruskal–Wallis test followed by a Dunn’s post-hoc test. The p-values are indicated in the graphs: *p < 0.05, and **p < 0.01, ***p < 0.001. Bars: (**A**) 100 µm.

In the DCX reporter model, the numbers of GFP^+^ cells which were also labeled by Olig2 or Olig2 and CC1 were analyzed after 6 weeks of cuprizone treatment (demyelination group) which were followed by 2 weeks of normal chow (remyelination group) and compared to controls (Fig. 5A). In the GL plus SGZ, the numbers of GFP^+^Olig2^+^ cells were significantly increased after 2 weeks of remyelination (Fig. 5B), whereas no significant effect was observed in the hilus (Fig. 5C). More pronounced was the increase of the GFP-expressing portion of Olig2^+^ cells during remyelination in both regions (trend for hilus p = 0.0908) (Fig. 5D,E). The number of cells labeled by the DCX reporter which also had a marker profile of mature oligodendrocytes, i.e. GFP^+^Olig2^+^CC1^+^, was significantly increased during de- and remyelination in both subregions of the DG analyzed (Fig. 5F,G). The GFP-expressing portion of mature oligodendrocytes expanded as well in both regions during de- and remyelination (Fig. 5H,I). Neither the number of GFP^+^ OPCs (Olig2^+^CC1^−^) nor the size of the GFP-expressing percentage of OPCs were significantly affected by de- or remyelination in either region (Fig. 5J–M). Taken together, these results indicate that the cuprizone-induced loss of oligodendrocytes induces an increased differentiation of DCX^+^ NPCs to mature oligodendrocytes in the hippocampal DG.

The analysis of oligodendroglial markers in the posterior part of the corpus callosum (CC) in the DCX reporter model, showed similar patterns, i.e. a significant increase of OPCs and mature oligodendrocytes, as well as DCX reporter-labeled mature oligodendrocytes but not OPCs after demyelination. However, the time course was different, as cell numbers reached a peak after 6 weeks of cuprizone treatment which was followed by a return to control levels (Suppl. Fig. 1).

### Increased percentage of BrdU^+^GFP^+^ NPCs and more BrdU^+^GFP^+^Olig2^+^ cells during remyelination

**Figure 6:**
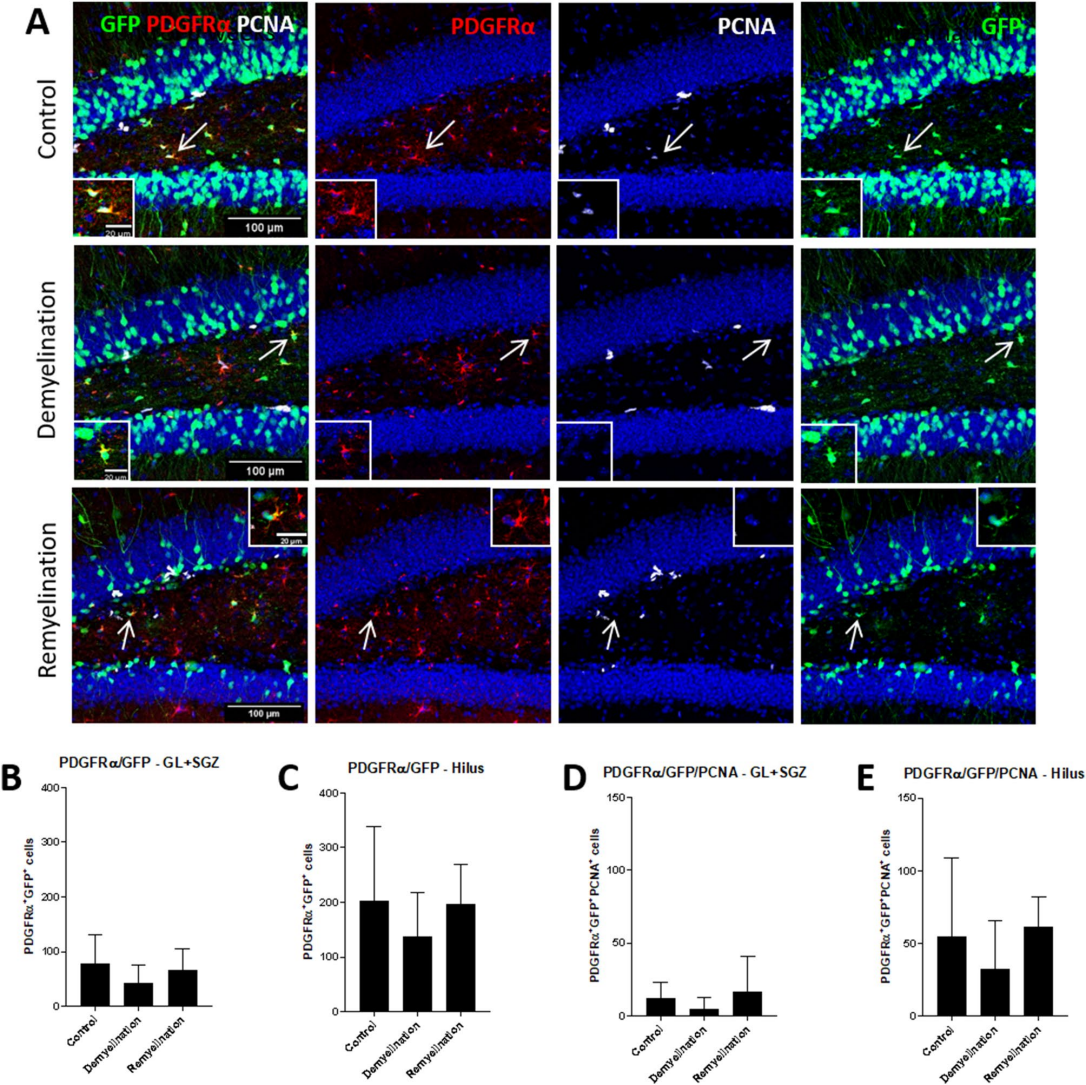
Number of GFP^+^PDGFRα^+^ cells not affected by de- or remyelination. (**A**) Triple-labeling of GFP (green), PDGFRα (red), and PCNA (white). The GFP^+^PDGFRα^+^PCNA^+^ (control) or GFP^+^PDGFRα^+^ (de- and remyelination group) cells indicated by arrows are magnified in inserts. (**B**,**C**) The numbers of GFP^+^PDGFRα^+^ cells were not significantly altered by de- or remyelination in the (**B**) GL plus SGZ or the (**C**) hilus. (**D**,**E**) Also the number of PCNA^+^ proliferating GFP^+^PDGFRα^+^ cells were not significantly affected in the (**D**) GL plus SGZ or the (**E**) hilus of the DG. Values are depicted as means + S.D. (controls and demyelination: n = 8 per group; remyelination: n = 7). Statistical significance was evaluated using (**B**,**C**,**E**) a one-way ANOVA followed by a Tukey post-hoc test or (**D**) a Kruskal–Wallis test followed by a Dunn’s post-hoc test. No significant differences were found (at the p < 0.05 level). Bars: (**A**) 100 µm.

**Figure 7:**
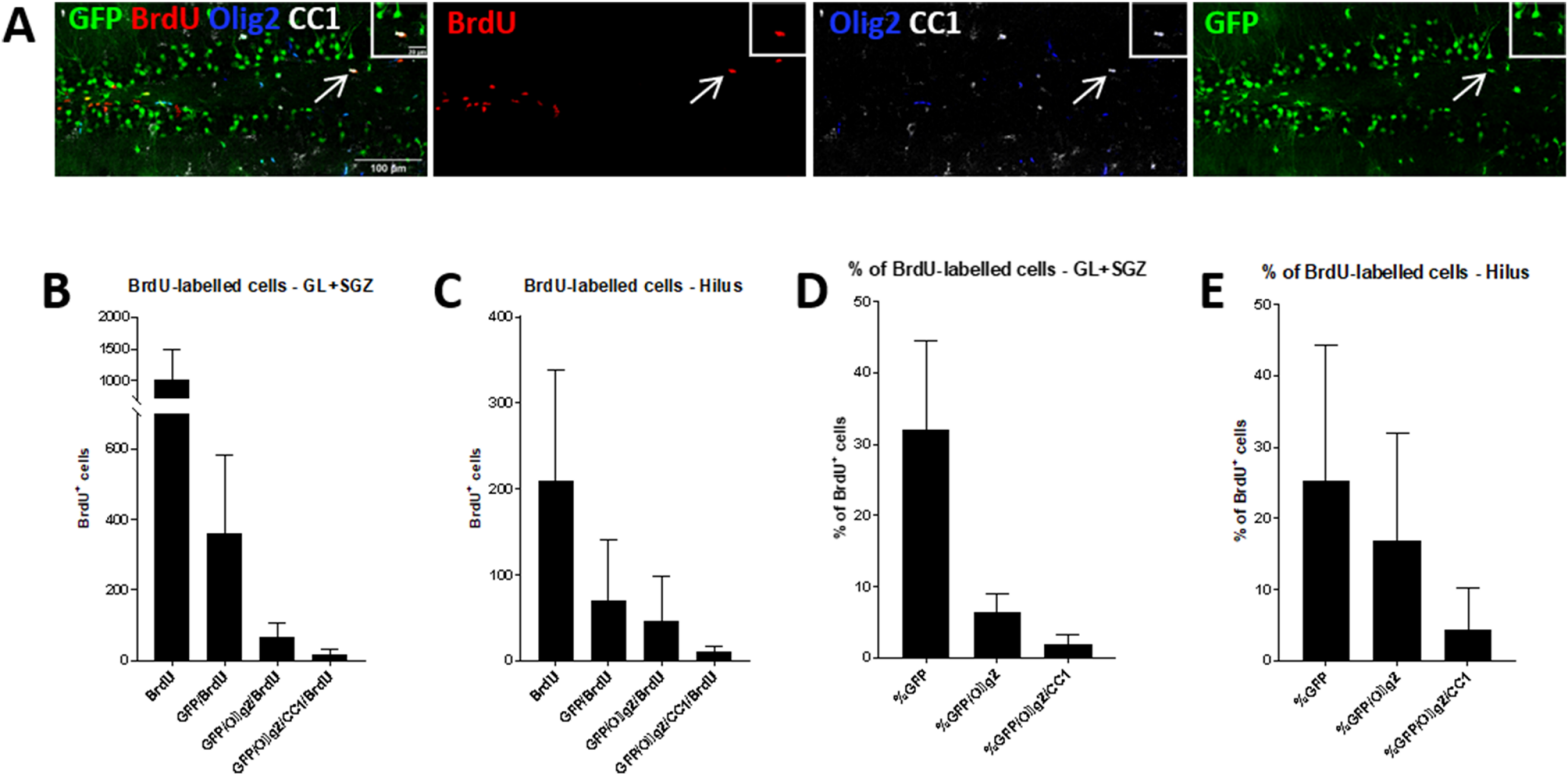
BrdU-labeled DCX reporter GFP^+^ mature oligodendrocytes 1 week after tamoxifen induction. (**A**) Quadruple-labeling of GFP (green), BrdU (red), Olig2 (blue), and CC1 (white) in the 1-week control group (10 days after the last TAM administration and 3 days after the last BrdU injection). The GFP^+^Olig2^+^CC1^+^BrdU^+^ cell indicated by the arrows is magnified in the inserts. (**B**–**E**) Analysis of the different BrdU^+^GFP^+^ cell populations directly after tamoxifen induction (control group 1 week; n = 5). The total numbers of BrdU^+^, GFP^+^BrdU^+^, GFP^+^Olig2^+^BrdU^+^ and GFP^+^Olig2^+^CC1^+^BrdU^+^ cells in the 1-week control group are shown for (**B**) GL plus SGZ and (**C**) the hilus of the DG. The percentages of BrdU^+^ cells in which also GFP, GFP/Olig2 or GFP/Olig2/CC1 are expressed in the (**D**) GL plus SGZ or (**E**) hilus. Values are depicted as means + S.D. Bars: (**A**–**C**) 100 µm, insert 20 µm.

**Figure 8:**
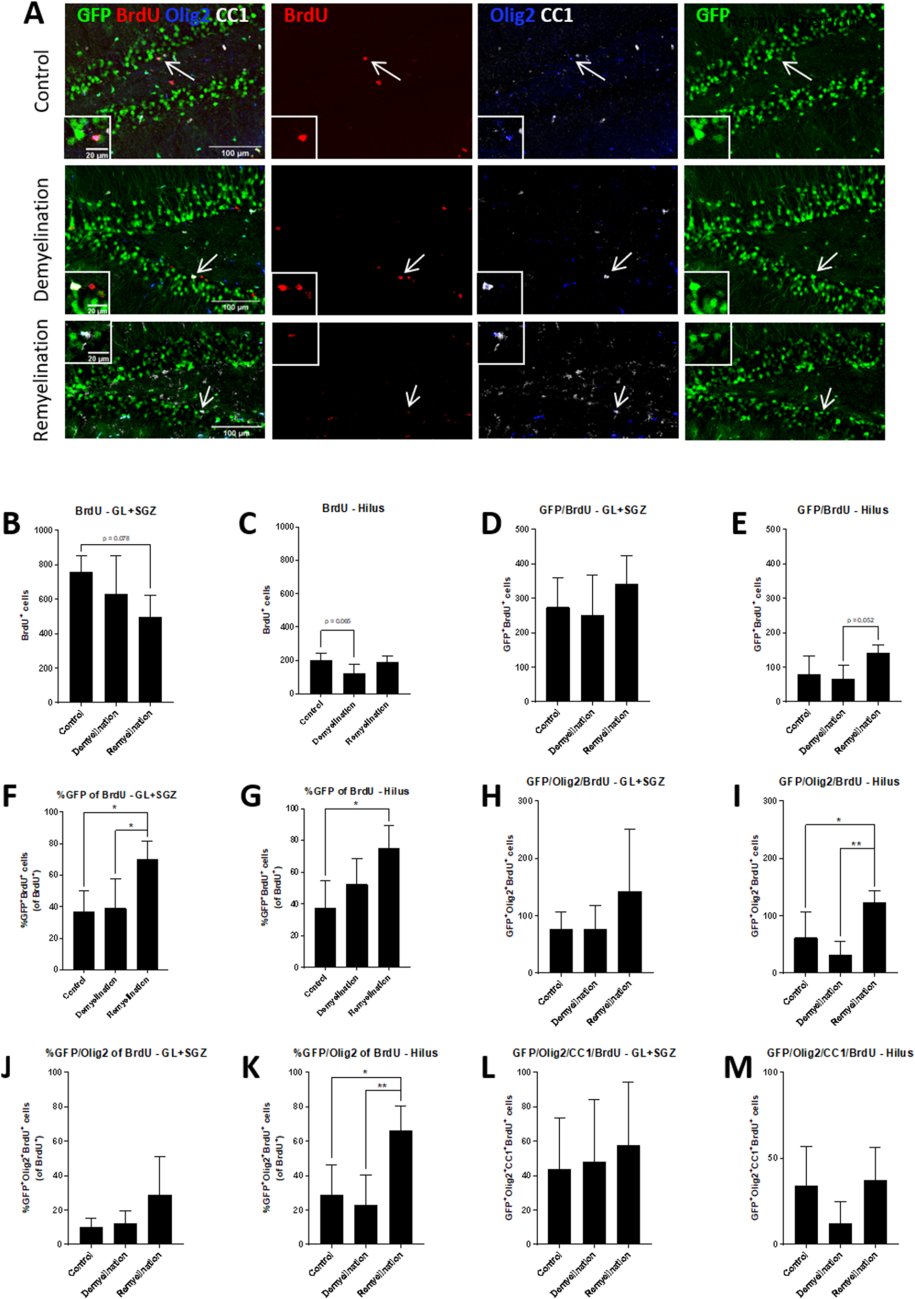
Higher portion of BrdU^+^GFP^+^ NPCs and more BrdU^+^GFP^+^Olig2^+^ cells during remyelination. (**A**) Quadruple-labeling of GFP (green), BrdU (red), Olig2 (blue), and CC1 (white). GFP^+^Olig2^+^BrdU^+^ (control) or GFP^+^Olig2^+^CC1^+^BrdU^+^ cells (de- and remyelination groups) indicated by arrows are magnified in the inserts. (**B**,**C**) Total numbers of BrdU^+^ cells in the control, de- and remyelination group. When comparing controls and the demyelination group, which were both terminated at the same time, there was a trend for a lower number of BrdU^+^ cells only in the hilus (p = 0.065). The remyelination group, which received BrdU at the onset of cuprizone withdrawal and which survived for 1 week longer after BrdU administration than the other two groups, had a trend for fewer BrdU^+^ cells in comparison to the controls only in the GL plus SGZ (p = 0.078). (**D**,**E**) There was a trend for an increase of BrdU^+^GFP^+^ cells in the remyelination group only in the hilus (p = 0.052 in comparison to the demyelination group), but (**F**,**G**) the percentage of BrdU-labeled GFP^+^ cells significantly increased in both regions in comparison to controls (in the GL plus SGZ also compared to the demyelination group). (**H**–**K**) Both numbers (**H**,**I**) and percentages (**J**,**K**) of BrdU-labeled GFP^+^Olig2^+^ cells were highest in the remyelination group, this, however, was only significant in the hilus. (**L**,**M**) The numbers of BrdU-labeled GFP^+^Olig2^+^CC1^+^ mature oligodendrocytes were not significantly affected by de- or remyelination. Values are depicted as means + S.D. (controls and demyelination: n = 5 per group; remyelination: n = 4). Statistical significance was evaluated using (**B**–**I**, **K**–**M**) a one-way ANOVA followed by a Tukey post-hoc test or (**J**) a Kruskal–Wallis test followed by a Dunn’s post-hoc test. The p-values are indicated in the graphs: *p < 0.05, **p < 0.01. Bars: (**A**) 100 µm, inserts 20 µm.

The number of GFP^+^ cells expressing PDGFRα, a marker which is often used to identify OPCs, was not significantly changed by de- or remyelination (Fig. 6A–C), and also the proliferation of these cells was not significantly altered (Fig. 6A,D,E). To further analyze proliferation and subsequent survival as well as oligodendroglial fate of GFP^+^NPCs and OPCs in the DCX reporter model, we administered BrdU on 5 consecutive days. In the 1-week control group, we found BrdU-labeled GFP^+^, GFP^+^Olig2^+^ as well as GFP^+^Olig2^+^CC1^+^ cells (in order from most frequent to least) in both analyzed regions (Fig. 7A–E). After cuprizone treatment (Fig. 8), there was, only in the hilus, a trend for a reduction (p = 0.065) in the number of BrdU^+^ cells between the demyelination group and the controls, which both survived for 3 days after the last BrdU injection (Fig. 8B,C). In contrast, the remyelination group, which received the first dose of BrdU on the day when the cuprizone treatment was stopped and then survived for 14 more days, showed a trend (p = 0.078) for fewer BrdU^+^ cells in comparison to the controls only in the GL plus SGZ (Fig. 8B,C). Despite the longer survival time, the number of BrdU-labeled GFP^+^ cells was highest in the remyelination group in both subregions (GL plus SGZ not significant, hilus: p = 0.052 in comparison to the demyelination group) (Fig. 8D,E). Interestingly, the portion of BrdU-labeled cells which was GFP^+^ significantly increased in the remyelination group in the GL plus SGZ in comparison to both the controls and the demyelination group, and in the hilus in comparison to controls (Fig. 8F,G). Concerning the oligodendroglial fate of GFP^+^ NPCs, results show that both numbers and percentages of BrdU-labeled GFP^+^Olig2^+^ cells were highest after remyelination, but this was significant only in the hilus (Fig. 8H–K). The numbers and percentages of BrdU-labeled GFP^+^Olig2^+^CC1^+^ were not significantly altered by de- or remyelination, but the total numbers of these quadruple-labeled cells were very low (Fig. 8L,M, percentages not shown, because the pattern was similar to the absolute numbers).

These results indicate that the increase in GFP^+^ NPC-derived mature oligodendrocytes observed in the DG might be, at least partially, due to an increased proliferation of GFP^+^ and GFP^+^Olig2^+^ NPCs which then further differentiate towards a more mature oligodendroglial phenotype.

## Discussion

### Do hippocampal NPCs contribute to new oligodendrocytes after myelin damage?

The aim of the present study was to investigate whether DCX^+^ NPCs in the hippocampus are able to replace lost cells of the oligodendroglial lineage and thereby contribute to myelin repair.

There is evidence from different demyelination models that oligodendrocytes can be generated by the SVZ neurogenic niche. This is the case for focal demyelination models^33–35^ in which DCX^+^ cells migrating to the corpus callosum were found^30^. Fate mapping of DCX-expressing NPCs in a focal demyelination model showed that some oligodendrocytes in the corpus callosum were generated from a subset of adult SVZ progenitors after demyelination (interestingly, this occurred by differentiation rather than proliferation)^31^. A fate mapping analysis of GFAP-expressing cells in a focal demyelination model identified oligodendroglial cells which were derived from the SVZ. However, these cells were not positive for MBP and, therefore, it was concluded that they do not contribute to myelination^36^. After cuprizone-induced demyelination, SVZ-derived myelinating oligodendrocytes have been described in subcortical white matter lesions^37^ and the corpus callosum^38,39^. However, another study in the cuprizone model reported an increased migration of DCX^+^ cells from the SVZ only towards the olfactory bulb, but not to lesion sites^40^.

Within the hippocampal neurogenic niche, oligodendrogenesis has only been described so far as a result of interventional strategies (reviewed in^41^). For example, hippocampal NPCs have changed their cell fate from neuronal to oligodendroglial after retroviral-mediated overexpression of the bHLH transcription factor Ascl1 (Mash1)^42^, inactivation of neurofibromin 1^43^, deletion of *Nfix*^44^ and after stimulation with mesenchymal stem cell-derived factors^45,46^.

### Does cuprizone-induced demyelination alter hippocampal adult neurogenesis?

In the present study, two regions analyzed in the hippocampal DG had significantly lower levels of MBP after cuprizone treatment. In the hilus, which has more myelinated fibers, the changes in myelination were more pronounced than in the GL plus SGZ. This is in accordance with other studies describing that the hippocampus is demyelinated in the cuprizone model^23–28^. A significant decrease of oligodendroglial lineage cells, and in particular mature oligodendrocytes, was observed after demyelination in both analyzed subregions of the DG. By the second week of remyelination, the numbers of these cells were restored. Particularly, in the GL plus SGZ the numbers of mature oligodendrocytes after remyelination exceeded control levels. This was remarkable since this region is not only less myelinated, but we also found fewer (proliferating) OPCs in this region than in the hilus.

In the posterior CC, numbers of oligodendroglial cells drastically increased already after 6 weeks of cuprizone treatment and returned to control levels after 2 weeks of remyelination (for CC data see supplemental material). These results are similar to those of a study by Baxi and colleagues in which a significant reduction of CC1^+^ cells in the CC was observed after 4 weeks of cuprizone treatment. However, the number of these cells recovered quickly to control levels after 2 more weeks of continued cuprizone treatment, and further increased during the remyelination period. In addition, the numbers of PDGFRα^+^ OPCs were higher than in controls during the entire study period. The same study found that the hippocampus, and in particular CC1^+^ oligodendrocytes, recovered more slowly from cuprizone-induced demyelination^27^. These results suggest that demyelination leads to an increased mobilization and differentiation of OPCs than under control conditions, but the timing and extent are region-specific.

The effect of cuprizone treatment on hippocampal neurogenesis has not been extensively studied. In the present study, the demyelination group had the lowest number of DCX^+^ cells, this reduction was, however, not significant in comparison to the other groups. In contrast to our results showing no significant differences in the numbers of DCX^+^ cells, reduced numbers of DCX^+^ NPCs were reported in a 4-week^47^, and a 6-week^48^ cuprizone model. Additionally, and Zhang and collaborators described a reversible loss of DCX staining after a 6-week treatment with 0.3% cuprizone alone or in combination with rapamycin^49^. These, seemingly, contradictory findings might be in part explained by differences in analysis methods and in cuprizone models, but this would need to be further investigated. Additionally, we did not find a significant difference in the proliferation of DCX^+^ cells.

### DCX-based fate mapping of NPCs reveals oligodendroglial potential in the adult hippocampus

Fate mapping analysis in DCX-CreER^T2^/Flox-EGFP mice revealed a small number of GFP^+^Olig2^+^CC1^+^ cells in healthy controls. This suggests that, even under physiological conditions, a small percentage of mature oligodendrocytes in the hippocampal DG is derived from DCX-expressing NPCs. Others have recently demonstrated that de novo myelination in hippocampal-cortical networks plays an important role in spatial learning and memory consolidation^50^. It would be interesting to investigate if hippocampal NPC-derived mature oligodendrocytes are involved in these processes. Ten days after induction of the DCX reporter, the number of GFP^+^ NPC-derived mature oligodendrocytes was slightly higher (GL plus SGZ: 35 ± 18, hilus: 27 ± 6) than 5 weeks later (GL plus SGZ: 11 ± 17, hilus: 18 ± 13). This might suggest that some of these mature oligodendrocytes which were derived from cells with an active DCX reporter did not survive this 6-week period under control conditions. While the numbers of GFP^+^Olig2^+^CC1^+^ mature oligodendrocytes were comparable in both DG subregions analyzed, the extent of co-expression of other oligodendroglial markers differed greatly. In the hilus, the majority of DCX-expressing NPCs also expressed Olig2, or other oligodendroglial markers. In the GL plus SGZ, however, the percentage of these NPCs with oligodendroglial features was very small. This might indicate that different NPC subpopulations reside in the two subregions of the DG. However, the ability of the NPCs in both regions to generate mature oligodendrocytes seems to be similar, not only under control conditions, but also during remyelination. A co-expression of DCX and markers for OPCs has also been described by others. Boulanger and Messier reported a co-expression of DCX and PDGFRα, and GFP(NG2) in NG2-CreER:EYFP reporter mice, but they found no co-labeling with GST pi, a marker for mature oligodendrocytes^51^.

Analyzing the numbers of DCX reporter GFP^+^ OPCs and mature oligodendrocytes after 6 weeks of cuprizone treatment in the hippocampal DG, we observed an opposite trend than for OPCs and mature oligodendrocytes in general. Here, we found that the numbers of NPC-derived GFP^+^ OPCs remained stable, while a pronounced increase of GFP^+^ mature oligodendrocytes occurred already during demyelination (which was sustained until 2 weeks of remyelination). Also, the DCX reporter GFP^+^ portion of all Olig2^+^CC1^+^ mature oligodendrocytes was markedly higher after cuprizone treatment and in the remyelination group. This suggests that the very low basal levels of oligodendroglial differentiation of DCX-expressing NPCs are increased in response to the cuprizone-induced loss of oligodendrocytes. The number of NPC-derived mature oligodendrocytes was similar in both DG subregions analyzed. This indicates that NPCs of GL plus SGZ and hilus seem to have a comparable oligodendrogenic potential, even though the percentage of NPCs co-expressing oligodendroglial markers differed markedly under control conditions. Additionally, it might be hypothesized that NPC-derived oligodendroglial cells in the hippocampus might be less vulnerable to cuprizone intoxication than other oligodendroglia.

In the posterior CC, the DCX reporter GFP^+^ subset of mature oligodendrocytes followed the same trend as the total numbers of this cell type (both were markedly increased after 6 weeks of cuprizone treatment). These results are similar to those reported by Brousse et al., who found, using fate mapping in NestinCreERT2-YFP and PDGFRaCreERT2-YFP mice, a massive increase of SVZ-derived NPCs in the CC after 5 weeks of cuprizone treatment and, moreover, that these cells adopted an oligodendrocytic fate^38^. The differences in cell dynamics that we observed between the hippocampus and the CC might be explained by the faster recovery of this white matter region and, in particular of its mature oligodendrocytes, from cuprizone-induced effects, which has been previously demonstrated^27^. It is possible that a differential effect of cuprizone intoxication on NPC-derived and other oligodendroglial cells may have manifested at an earlier time point in the CC. There is increasing evidence that oligodendroglial lineage cells are heterogeneous in their differentiation and proliferation capacity, for example between white and grey matter (reviewed in^10^), which might explain some of the differences we observed between the regions analyzed.

### Is the increase in GFP^+^ hippocampal NPC-derived mature oligodendrocytes due to increased proliferation or differentiation?

The analysis of the proliferation of DCX reporter GFP^+^ OPCs (positive for PDGFRα) did not show significant differences during de- or remyelination. For this reason, we used BrdU-labeling to further analyze proliferation and subsequent survival of GFP^+^ NPCs. We found a low number of BrdU-labeled GFP^+^ mature oligodendrocytes (Olig2^+^CC1^+^) under all investigated conditions. This indicates that a small number of NPC-derived mature oligodendrocytes is continuously being generated in the hippocampal DG. During remyelination, the numbers of BrdU-labeled GFP^+^ and GFP^+^Olig2^+^ cells increased, which was more pronounced in the hilus than in the GL plus SGZ. This suggests that the generation of NPC-derived mature oligodendrocytes accelerates in response to demyelination. However, higher proliferation alone might not fully explain the observed increase in the number of GFP^+^Olig2^+^CC1^+^ cells, which was already significant at the end of the demyelination period when the number of BrdU-labeled cells was not markedly changed. A fate mapping analysis of DCX-expressing NPCs of the adult SVZ in a focal demyelination model revealed that some oligodendrocytes in the CC (i.e. GFP^+^ cells that expressed CC1 and CNP) were generated from a subset of adult SVZ progenitors after demyelination by differentiation instead of proliferation^31^. It is possible that a similar mechanism of a higher rate of oligodendroglial differentiation of GFP^+^ NPCs is responsible for the earlier increase in NPC-derived mature oligodendrocytes. This, however, would need further investigation.

## Conclusion

Our results indicate that the potential for cell lineage plasticity is present in a subpopulation of hippocampal NPCs that is usually thought to be committed to a neuronal fate, and that this process is enhanced after demyelination. However, if these oligodendrocytes derived from DCX-expressing NPCs actively contribute to axonal myelin sheaths still needs to be determined.

## Supplementary information

Supplementary Figure 1.

## Data Availability

The datasets analyzed during the current study are available from the corresponding author on reasonable request.
