# Supplementary material for: DCX+ neuronal progenitors contribute to new oligodendrocytes during remyelination in the hippocampus
Source: Sci Rep. 2020 Nov 18;10:20095. doi: 10.1038/s41598-020-77115-w (PMC7674453; doi:10.1038/s41598-020-77115-w)
Supplement: Supplementary file 1 — Supplementary Figure 1. [file 41598_2020_77115_MOESM1_ESM.pdf]

Supplementary information:

DCX$^+$ neuronal progenitors contribute to new oligodendrocytes during remyelination in the hippocampus

Barbara Klein[1,2*], Heike Mrowetz[1,2], Christina Kreutzer[2,3], Peter Rotheneichner[2,3], Pia Zaunmair[2,3], Simona Lange[1,2], Roland Coras[4], Sebastien Couillard-Despres[2,3,5,8], Francisco J. Rivera,[1,2,6,7,8], Ludwig Aigner[1,2,5,8].

[1]Institute of Molecular Regenerative Medicine, Paracelsus Medical University, Salzburg, Austria

[2]Spinal Cord Injury and Tissue Regeneration Center Salzburg (SCI-TReCS), Paracelsus Medical University, Salzburg, Austria

[3]Institute of Experimental Neuroregeneration, Paracelsus Medical University, Salzburg, Austria

[4]Department of Neuropathology, Universitätsklinikum Erlangen, Friedrich-Alexander University Erlangen-Nürnberg (FAU), Erlangen, Germany

[5]Austrian Cluster for Tissue Regeneration, Vienna, Austria

[6]Laboratory of Stem Cells and Neuroregeneration, Institute of Anatomy, Histology and Pathology, Faculty of Medicine, Universidad Austral de Chile, Valdivia, Chile

[7]Center for Interdisciplinary Studies on the Nervous System (CISNe), Universidad Austral de Chile, Valdivia, Chile

[8]These authors jointly supervised this work: Sebastien Couillard-Despres, Francisco J. Rivera and Ludwig Aigner

[*]email: barbara.klein@outlook.com

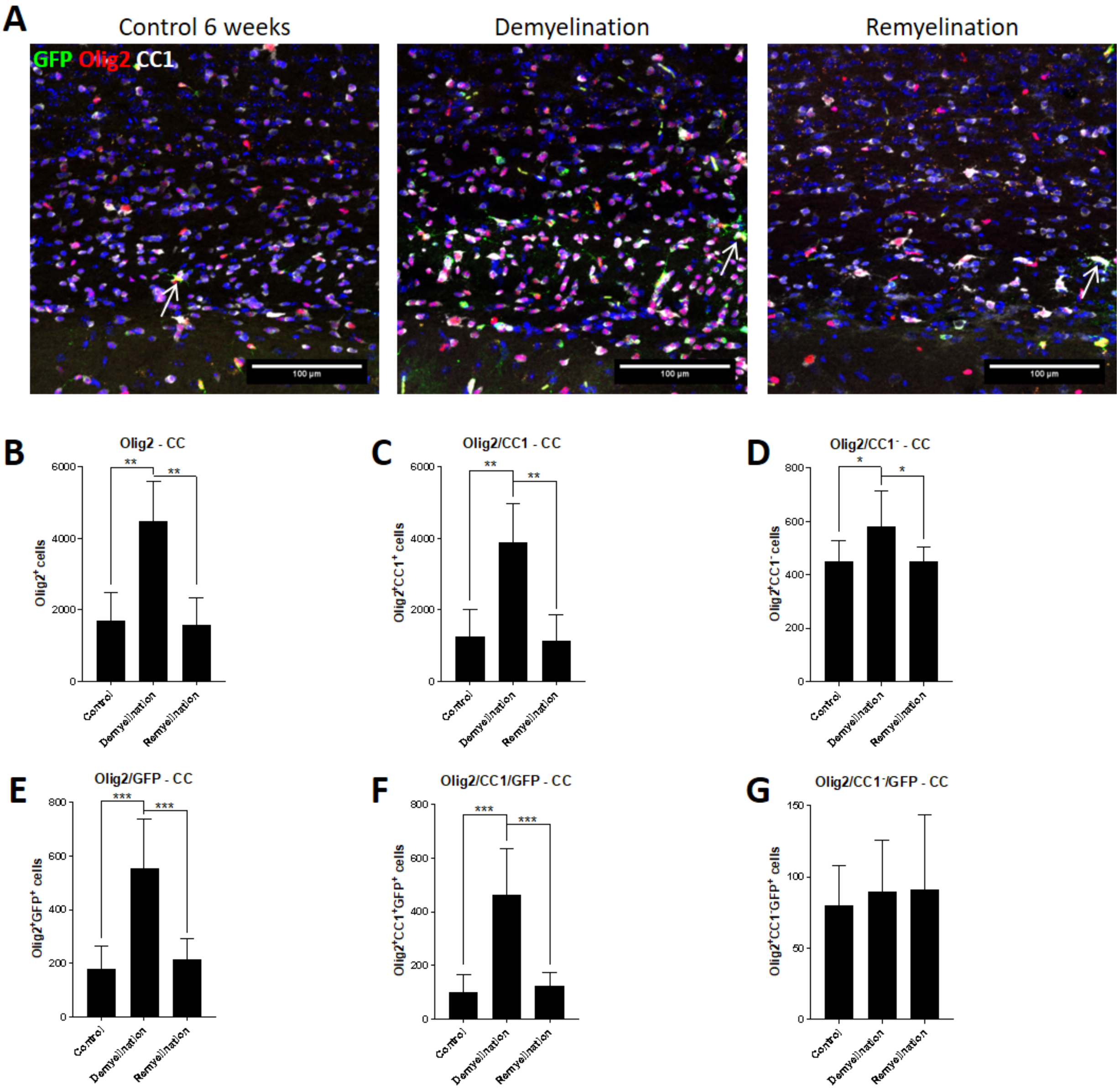

Suppl. Figure 1 Increased number of DCX reporter GFP$^+$ mature oligodendrocytes after cuprizone-induced demyelination in the corpus callosum (CC). (A) Triple-labeling of GFP (green), Olig2 (red) a marker for oligodendrocyte lineage cells, and CC1 (white) which is expressed by mature oligodendrocytes in controls, the demyelination group, and after 2 weeks of remyelination. GFP$^+$Olig2$^+$CC1$^+$ cells are indicated by arrows. (B-F) There was a significant increase of (B) Olig2$^+$ cells, (C) Olig2$^+$CC1$^+$ mature oligodendrocytes and (D) Olig2$^+$CC1$^-$ OPCs, as well as (E) GFP$^+$Olig2$^+$ cells, and (F) GFP$^+$Olig2$^+$CC1$^+$ mature oligodendrocytes after demyelination in the CC. (G) The number of GFP$^+$Olig2$^+$CC1$^-$ OPCs did not change significantly during de- and remyelination. Values are depicted as means + S.D. (controls and demyelination: n=8 per group; remyelination: n=7). Statistical significance was evaluated using (D-F,) a one-way ANOVA followed by a Tukey post-hoc test or (B,C,G) a Kruskal-Wallis test followed by a Dunn's post-hoc test. The p-values are indicated in the graphs: *$p < 0.05$, **$p < 0.01$, and ***$p < 0.001$. Bars: (A) 100 µm.
